# EXPLAINING DIFFERENCE: LATINX AND STRUCTURAL VULNERABILITY IN ADRD

**DOI:** 10.1093/geroni/igae098.2143

**Published:** 2024-12-31

**Authors:** Ignacia Arteaga, Alma Hernández de Jesús, Brandi Ginn, Corey Abramson, Daniel Dohan

**Affiliations:** University of California San Francisco, San Francisco, California, United States; University of California San Francisco, San Francisco, California, United States; University of California San Francisco, San Francisco, California, United States; Rice University, Tucson, Arizona, United States; University of California San Francisco, San Francisco, California, United States

## Abstract

Latinos/as/x (hereafter Latinx) residing in the U.S. are one and a half times more likely than non-Latinx white residents to develop cognitive impairment during their lives. Despite significant heterogeneity in nativity, language, socio-economic status, geographic region of residence, culture, and other factors, US-resident Latinx face shared structural barriers to health that may help explain inequities in incidence, time of diagnosis, and health outcomes of Alzheimer’s Disease and Related Dementias (ADRD). This work uses comparative ethnography to unpack the everyday experiences of Latinx families, including people living with dementia (PLWD) and ADRD. We focus on how PLWD and their caregivers negotiate family dynamics informed by structural vulnerabilities. We draw on 84 in-depth interviews with older adults and their family caregivers alongside three years of participant observation across community centers in California and Maine. We document how ‘familism,’ a cultural orientation understood as the strong reliance on close kin relations to look after the ill, is brought into tension when ADRD, structural vulnerability, and multi-morbidities among family members are at stake. When facing limited access to healthcare and social support, family caregiving and its monetary compensation can become a source of within-family tension and even dispute. This may be especially true for Latinx families for whom caregiving income constitutes an important part of their everyday income-generating strategies. These findings offer an opportunity to reflect on current policy efforts to support structurally marginalized families living with ADRD in the US.

